# Cognitive Recovery After Poststroke Delirium (RECOVER): Protocol for a Longitudinal Multimodal Study on Cognitive Assessment in Patients With Stroke

**DOI:** 10.2196/77508

**Published:** 2026-01-12

**Authors:** Lena S Geiger, Sebastian Klütz, Christian Mychajliw, Christoph Gäbele, Andreas Jooß, Constanze Single, Katharina Feil, Ulf Ziemann, Annerose Mengel

**Affiliations:** 1 Department of Neurology & Stroke, University Hospital Tübingen, Hoppe-Seyler-Street 3, Tübingen, 72076, Germany, 49 070712961675; 2 Hertie Institute for Clinical Brain Research, University of Tübingen, Tübingen, Germany; 3 Geriatric Center, University Hospital for Psychiatry and Psychotherapy, University of Tübingen, Tübingen, Germany; 4 TuCAN, Tübingen Cognitive Assessment for Neuropsychiatric Disorders, Tübingen, Germany

**Keywords:** cognitive assessment, cognitive functioning, cognitive outcome, fMRI, functional magnetic resonance imaging, longitudinal design, memory functions, multimodal approach, poststroke cognitive impairment, poststroke delirium, stroke

## Abstract

**Background:**

Poststroke delirium (PSD) is a severe complication in patients with acute stroke and is characterized by rapid onset fluctuating symptoms, which affect multiple domains (cognition, motor system, and sleep-wake cycle). Similar to other types of delirium, PSD is associated with longer hospitalization, higher mortality, and a higher disability rate. Behavioral studies on cognitive functioning showed significantly poorer cognitive outcomes in both patients with and without PSD compared to healthy controls. Thus, the distinction between “stroke-related” and “PSD-related” cognitive impairments remains unclear. A frequently affected and highly disabling cognitive domain is memory function. However, imaging studies, particularly task-based functional magnetic resonance imaging (MRI) studies on PSD, are currently scarce and represent a significant gap in the existing literature.

**Objective:**

This longitudinal proof-of-concept study aims to investigate the short- and long-term effects of stroke and PSD on cognitive outcome using a multimodal approach and to outline a protocol for a repeated multimodal cognitive assessment in patients with stroke.

**Methods:**

We developed a longitudinal study protocol to investigate short- and long-term effects of stroke and PSD on cognitive impairment and recovery. Poststroke cognitive impairment (PSCI) will be assessed in a comprehensive digital multimodal approach including a multidomain neuropsychological app to facilitate a standardized, rapid testing, particularly for long-term outcomes, as well as task-based functional MRI (introducing a modified working memory task) during the acute (prior PSD development) and postacute phase post stroke and at a 3-month follow-up. In total, 40 patients with acute stroke, divided into a PSD group and non-PSD group (control), will be examined. In the context of the proof-of-concept study, the eligibility of a modified working-memory task, an app-based neuropsychological assessment, and a multimodal MRI protocol will be evaluated. The primary endpoint is a between-group comparison of the cognitive outcome, defined as global PSCI at a 3-month follow-up. Global PSCI will be classified as normal cognitive functioning, or mild, moderate, or severe cognitive impairment, according to the performance level (norm-referenced *z* scores) on a multidomain neuropsychological assessment.

**Results:**

This study was funded in December 2023. As of May 2025, 15 patients with stroke have been included. Recruitment and data collection were initiated in June 2024 and are ongoing until December 2025. The findings are expected to be published in summer 2026.

**Conclusions:**

This protocol outlines a proof-of-concept study aiming to fill a critical gap by systematically investigating cognitive functions in patients with acute stroke with and without delirium. Generating reliable preliminary data will provide essential groundwork for future large-scale research, ultimately enhancing understanding of the contribution of PSD to cognitive impairment.

## Introduction

Delirium is an acute clinical condition characterized by a wide range of symptoms affecting multiple domains. Patients with delirium often present with attention deficits, confusion, hallucinations, agitation, or disturbances in sleep-wake cycle. Typically, delirium occurs during critical illness, such as after surgery or stroke, with rapid onset and fluctuating symptoms [1]. Poststroke delirium (PSD) is one of the most common complications after stroke, with reported prevalence rates reaching up to 40% in older patients [1-3]. Both delirium in general and PSD in particular are associated with longer hospitalization, higher mortality, accelerated cognitive decline, and lower quality of life [1,4-11]. There is a broad consensus of a multifactorial origin [1,12,13], with predisposing factors such as dementia, age, comorbidities, and precipitating risk factors, including infection, stroke, or surgery [1,12,13]. The presence of multiple and fluctuating symptoms is diagnostically challenging, especially in patients with stroke with communication difficulties (eg, aphasia), and requires clinical expertise and standardized screening tools, such as the well-established ICDSC (Intensive Care Delirium Screening Checklist) [14,15]. Despite the high prevalence and the nondeclining mortality rate over the past decades [2,7], objective diagnostic tools and prognostic biomarkers (ie, fluid or proteomic biomarkers), as well as protective markers for long-term cognitive outcome, have not yet been implemented in clinical practice and remain the focus of ongoing studies [5,16-18].

In general, examining delirium patients is a challenging field, which limits the number of available studies. Patients with delirium, such as patients with PSD, represent a vulnerable and critically ill population. Especially during the acute (within 5 d poststroke) and the postacute phase (5 d to 12 wk poststroke), patients may experience secondary deteriorations or be scheduled for invasive diagnostic procedures or surgeries, making the conduction of studies difficult. Additionally, the fluctuating nature of delirium symptoms complicates the timing and consistency of study assessments. Furthermore, during the acute state of delirium, especially cases involving high arousal or hallucinating symptoms, cognitive assessment or magnetic resonance imaging (MRI) assessment becomes impossible, as patients cannot comply with task instructions. This results in a lack of studies and underscores the need for further research in this area. Another challenge in this field concerns the study design, varying with regard to concurrent versus sequential study designs, as well as the choice of an appropriate control group.

Previous studies have reported a higher risk of PSD in total anterior circulation infarcts compared to partial anterior, posterior, and lacunar infarcts [19-21]. Cortical networks in the anterior circulation, such as fronto-parietal networks, are known to be involved in higher-level cognitive functions, including executive functions, attention, and memory. These very cognitive domains are affected in PSD. Memory functions, which represent a critical component of human cognition, are frequently affected and represent a highly disabling cognitive domain in stroke survivors [22]. For example, memory impairments have been significantly associated with poor quality of life of patients with stroke and their caregivers, for example, [22,23], demonstrating the high relevance to daily life and potential burden. Moreover, memory assessment is a well-established component of both neuropsychological testing and in functional magnetic resonance imaging (fMRI) studies, making it an ideal focus for our study.

In the challenging field of cognitive assessment in patients with delirium, the majority of studies focus on behavioral measures including the assessment of multiple cognitive domains such as attention, language, memory, executive functions, and visuospatial construction. Previous studies have demonstrated a significantly poorer cognitive outcome (eg, global cognition and executive functions) in patients with postdelirium compared to healthy controls [24-26].

Additionally, advanced techniques such as MRI have been implemented to provide further insights. Structural MRI studies, which do not require a certain level of patients’ compliance, have reported abnormalities in white matter tracts of patients with postdelirium, which are known to be involved in memory and attention [1,13,27]. Since delirium is known to be a disorder of disrupted brain networks [1,12,16,28,29], fMRI studies, which were predominantly conducted as resting-state studies in patients with postdelirium, have demonstrated aberrant connectivity in dorsal prefrontal and subcortical regions compared to healthy controls, which may contribute to the emergence of delirium symptoms [13,28,30]. These findings in network dysfunctions are restricted to the default mode network, and memory functions may not be detected during resting-state fMRI [31]. An essential requirement for studying memory functions and related activity or connectivity dysfunctions in delirium is a memory task-based fMRI study. Unfortunately, these are exceedingly limited.

To date, only 1 fMRI study has been conducted, using a well-known N-Back working memory task [32]. While reporting no association between delirium and brain region activity, several methodological limitations and concerns have been raised, such as the infeasibility of the working memory task (especially at the time of discharge), the infeasible MRI protocol, as well as the absence of a control group. Of note, connectivity analyses have not been conducted. Further research is therefore needed, alongside the development of an adequate and reliable task for working memory assessment during fMRI.

An additional challenge in studying PSD and cognitive outcomes is the unresolved distinction between PSD-related and pure stroke−related cognitive impairments. In detail, cognitive deficits associated with PSD and other types of delirium, for example, attention or memory deficits, have also been reported in patients with stroke without PSD [23]. Previous imaging studies have shown reduced brain activation as well as abnormal connectivity within fronto-parietal networks in patients without PSD compared to healthy controls [31,33,34]. Thus, the origin of cognitive impairments in patients with PSD (ie, distinction between “stroke-related” vs “PSD-related” impairments) remains unclear.

To summarize, there is a lack of reliable and feasible fMRI tasks for assessing memory functions in patients with PSD. The few existing task-based fMRI studies have either been conducted without control groups or have only compared groups of patients with PSD to healthy controls, limiting the ability to disentangle the distinct cognitive and neural contributions of PSD versus stroke. Therefore, there is a significant gap in the literature regarding appropriate methods to study PSD and its cognitive outcomes, emphasizing the need for comprehensive, longitudinal investigations that combine neuropsychological assessments with functional neuroimaging.

To address this gap, we present a protocol for a comprehensive longitudinal proof-of-concept study that aims to systematically and comprehensively examine poststroke cognitive impairment (PSCI) in a critically ill stroke population, both with and without PSD (control group). Assessments will be conducted during the acute (within 5 d post stroke) and postacute phases (5‐14 d post stroke), and at a 3-month follow-up using a multicomponent neuropsychological assessment and task-based fMRI. Generating reliable feasibility and preliminary data through this approach will provide essential groundwork for future multicenter studies.

The specific objectives of this study are to (1) compare the global PSCI of patients with and without PSD at a 3-month follow-up, (2) compare domain-specific cognitive outcome measures of patients with and without PSD at a 3-month follow-up, (3) generate preliminary data regarding the differentiation between PSD-related and stroke-related cognitive impairment using behavioral and fMRI connectivity measures, and (4) contribute toward developing protective and prognostic tools for the early detection and treatment of PSD.

## Methods

### Study Design and General Procedures

**Figure 1.**
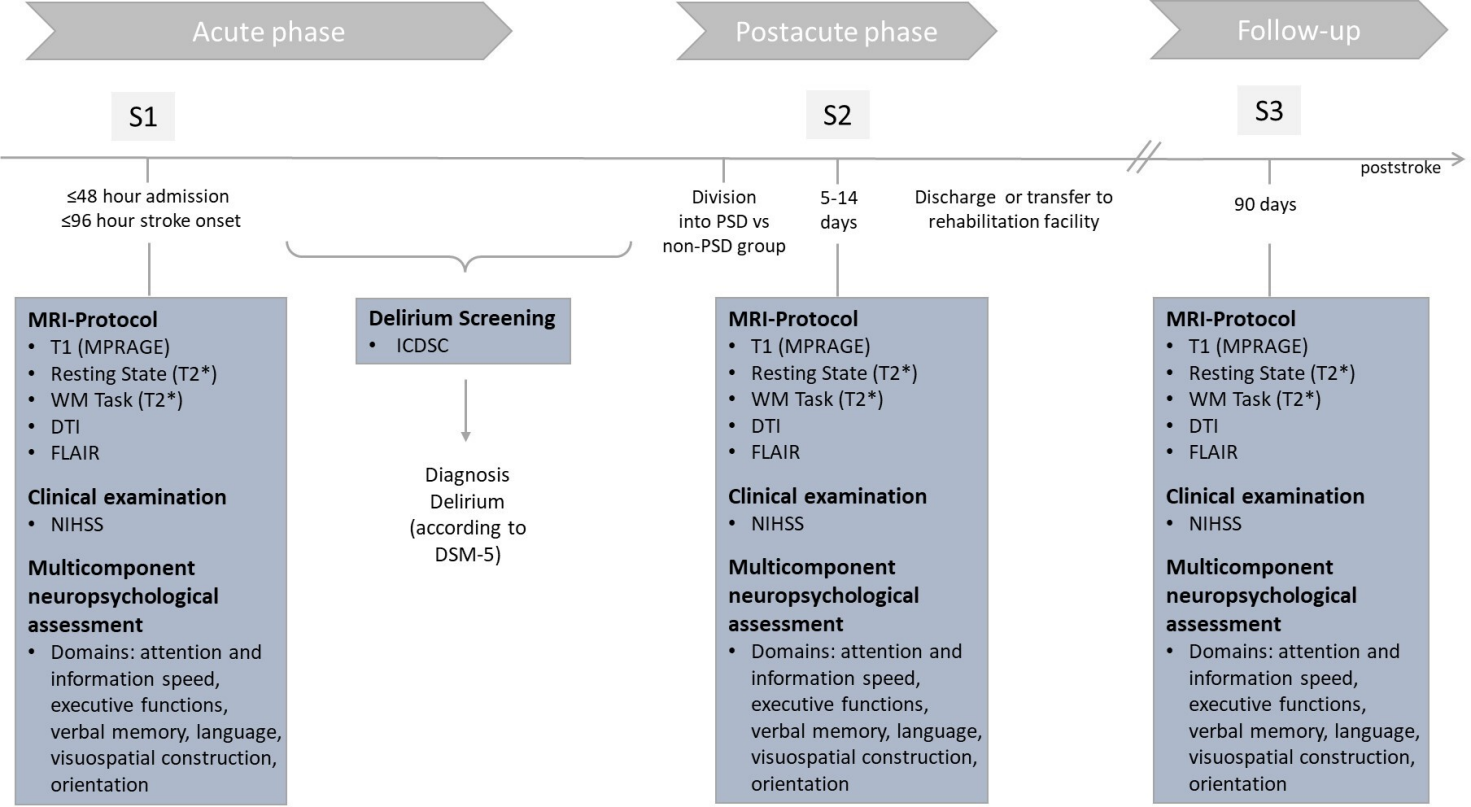
Overview of the RECOVER (Cognitive Recovery After Poststroke Delirium) study procedures. DSM-5: *Diagnostic and Statistical Manual of Mental Disorders, Fifth Edition*; DTI: diffusion tensor imaging sequence; FLAIR: fluid-attenuated inversion recovery sequence; ICDSC: Intensive Care Delirium Screening Checklist; MPRAGE: T1-weighted magnetization prepared rapid acquisition with gradient echoes sequence; MRI: magnetic resonance imaging; NIHSS: National Institutes of Health Stroke Scale; S1: session 1; S2: session 2; S3: session 3; T2*: T2-weighted echo planar imaging sequence; WM: working memory.

We present a protocol of the RECOVER (Cognitive Recovery After Poststroke Delirium) study, which is a monocenter study following a longitudinal design with 3 measurement points (S1-S3) and an individual trial duration of 3 months (Figure 1).

The three measurement points are as follows:

1. S1 during the acute phase poststroke (≤48 h after hospital admission; ≤96 h after stroke onset and before PSD development)
2. S2 during the postacute phase poststroke (5‐14 d poststroke, depending on delirium recovery)
3. S3 follow-up at 3 months poststroke (approximately 90 d poststroke)

Patients with stroke will be admitted to the Stroke Unit where a screening visit will be conducted, including a review of the inclusion and exclusion criteria. Following the review, written informed consent will be obtained from the patient or, if applicable, the legal guardian. Once consent has been obtained, the patient will be enrolled in the RECOVER study.

Each measurement (S1-S3) consists of a clinical examination, a multicomponent neuropsychological testing, and an MRI protocol. The MRI protocol is designed to acquire both structural (anatomical) data, as well as functional data (see the *Neuroimaging Protocol* section for more details). The clinical examination includes the National Institutes of Health Stroke Scale (NIHSS) [35] and will take approximately 15 minutes. The multicomponent neuropsychological assessment and the MRI scan (including positioning of patient) will each require 45‐60 minutes. A more detailed description is provided in the *Neuropsychological Assessment and Clinical Scores* section. S1 and S2 will be conducted as in-patient measurements, and the S3 3-month follow-up measurement will be conducted as an out-patient measurement.

During the course of the longitudinal study, in particular before S2, the cohort of patients with stroke will be subdivided into 2 groups, those with PSD and those without (non-PSD). Delirium screening using the ICDSC [32] will be conducted throughout hospitalization to identify the onset and duration of delirium. To ensure timely detection of delirium onset and recovery, assessments will be performed every 8 hours. A diagnosis will be made according to the DSM-5 criteria by an independent neurologist, who will review the clinical course alongside the ICDSC data. Delirium recovery is defined as 3 consecutive negative ICDSC screenings.

### Inclusion and Exclusion Criteria

This study includes patients with stroke with mild-to-moderate stroke symptoms (NIHSS ≤15; eg, [36,37]; Glasgow coma scale [GCS] >8). The prevalence of a PSD increases with age [20], reaching up to 40% among patients aged 60 years and older [1-3]. To ensure an equal distribution into the 2 study groups, only patients aged 60 years or older will be included in the RECOVER study. Textbox 1 gives an overview of all inclusion and exclusion criteria. When patients’ conditions or transfers preclude in-hospital assessment, follow-up visits at patients’ homes will be arranged.

Textbox 1. **Inclusion and exclusion criteria for the study enrollment.**

**Inclusion criteria**

- Aged ≥60 years
- National Institutes of Health Stroke Scale ≤15 [35]
- Acute ischemic stroke
- Stroke onset ≤96 hours
- Hospital admission ≤48 hours
- Informed consent by patient or legal guardian

**Exclusion criteria**

- Aphasia
- Reduced consciousness, defined as Glasgow Coma Scale ≤8 [38]
- Delirium onset before study enrollment
- Magnetic resonance imaging contraindication such as pacemakers or metal implants
- Preexisting conditions such as prior stroke, seizures, or traumatic head injury within the past 3 months, pre-existing neuropsychiatric disorders (eg, dementia, depression, and schizophrenia) or developmental delay
- High-grade paresis or plegia of the dominant hand

### Study Population and Recruitment

This longitudinal proof-of-concept study aims to include a total of 40 patients with acute stroke, equally distributed in a PSD (n=20) and a non-PSD group (n=20). All the patients with acute stroke will be recruited from the wards of the Neurological Department of the University Hospital Tübingen.

### Ethical Considerations

The study protocol has been approved by the local Ethics Committee of the Medical Faculty and the University Hospital Tuebingen (reference 444/2023BO2) and has been registered at ClinicalTrials.gov with the number NCT06680336. The study will follow the ethical principles for medical research involving human participants of the Declaration of Helsinki, adopted by the 18th General Assembly of the World Medical Association (World Medical Association, 1964). All patients or their legal guardians will provide informed consent before study enrollment.

This study protocol comes with important ethical considerations, given the vulnerability of patients with acute stroke, particularly those with PSD. Ethical responsibility and patient safety are central. To minimize the burden, neuropsychological testing and MRI sessions are kept as short as feasible and can be paused or rescheduled depending on the patient’s condition. Testing is stopped immediately if signs of distress or fatigue are observed. Clinical staff remain available throughout all procedures. We believe that including patients with cognitive vulnerability is ethically justified, as they are often underrepresented in research. However, their inclusion is balanced with stringent safeguards to ensure that participation does not place undue burden on their health or recovery. The data collected during the study will be pseudonymized to ensure the confidentiality of participants. All personal information will be treated with the utmost care and in accordance with applicable privacy regulations, including data protection laws. Patients are provided with a monetary compensation for their time and effort in accordance with the ethical guidelines of the study.

### Neuropsychological Assessment and Clinical Scores

We followed a multicomponent cognitive assessment strategy using standardized tests, questionnaires, and clinical scores in order to ensure a comprehensive assessment of PSCI. The selection of tests and domains aligns with the recommendations of major stroke organizations, such as the European Stroke Organisation and the Canadian Stroke Network [39,40]. The following domains were defined: attention and information speed, executive functions, verbal memory, language, visuospatial construction, and orientation (Table 1). In general, the tests, questionnaires, and clinical scores used in this study protocol are appropriate for our elderly study population, with available normative data. The order of test administration is standardized to ensure consistency across participants and is arranged to alternate between cognitive domains and varies in modality (eg, verbal vs visuospatial) to reduce cognitive load and prevent fatigue. To reduce practice effects across sessions, we use official parallel test forms when available. In consideration of the elderly study population, the selection of tests and questionnaires was also based on font size, or in cases where applicable, the font size was increased. Table 1 illustrates the questionnaires and clinical scores, as well as their targeted components used in this study. The neurological examination comprises the standardized NIHSS [35]. Some parts of the assessment will be digitalized using a tablet app called TuCAN (Tübingen Cognitive Assessment for Neuropsychiatric Disorders) [41] and a digitizer pencil. The TUCAN app was designed for elderly individuals and has previously been evaluated in healthy older populations [42,43]. Furthermore, it has been used to assess cognitive functions in 2 clinical populations: patients with cognitive impairment and patients with stroke [44,45]. To address potential unfamiliarity with digital technology in older patients with stroke, patients complete a short familiarization task before the formal assessment begins, during which they are asked to write a sentence or a few words on the tablet.

**Table 1.** Standardized tests, questionnaires, and clinical scores with the corresponding targeted domain.

Target domain and test/clinical score/questionnaire	S1^a^	S2	S3
Attention and processing speed			
TMT^b^—Part A [46]	✓	✓	✓
NAB^c^ Module Attention [47]	✓	✓	✓
Executive functions			
WM^d^ span within NAB Module Attention [47]	✓	✓	✓
TMT—Part B [46]	✓	✓	✓
Verbal memory			
MIS^e^ within MoCA^f^ [48]	✓	✓	✓
Language			
LIS^g^ within MoCA [48,49]	✓	✓	✓
Visuospatial construction			
VIS^h^ within MoCA [48,49]	✓	✓	✓
Orientation			
OIS^i^ within MoCA [48,49]	✓	✓	✓
Cognitive impairment			
MoCA [48]	✓	✓	✓
Questionnaires assessing cognitive functioning			
Self-rating CFQ^j^ [50]	✓		
Relative questionnaire: IQCODE^k^ [51]	✓		
Depression			
GDS^l^ [52]	✓	✓	✓
Quality of life			
SF^m^-12 [53]	✓		✓
Global disability			
(p)mRS^n^ [54]	✓		✓
Delirium screening			
ICDSC^o^ [55]	✓	✓	✓
Stroke symptoms			
NIHSS^p^ [35]	✓	✓	✓
Other variables			
Verbal intelligence test (MWT-B^q^ [56])	✓		
Demographics	✓		
Stroke location, medical interventions, comorbidities, etc	✓		

a S: session.

b TMT: trail making test.

c NAB: Neuropsychological Assessment Battery.

d WM: working memory.

e MIS: memory index scale.

f MoCA: Montreal Cognitive Assessment.

g LIS: language index scale.

h VIS: visuospatial index scale.

i OIS: orientation index scale.

j CFQ: cognitive failure questionnaire.

k IQCODE: Informant Questionnaire on Cognitive Decline in the Elderly.

l GDS: Geriatric Depression Scale.

m SF: Short Form health survey.

n (p)mRS: (pre)modified Rankin scale.

o ICDSC: Intensive Care Delirium Screening Checklist.

p NIHSS: National Institutes of Health Stroke Scale.

q MWT-B: German intelligence test *Mehrfach-Wortschatz-Intelligenztest*.

### Neuroimaging Protocol

Functional and structural MRI data will be acquired using a 3 Tesla Siemens Prisma whole-body imaging system (Siemens), equipped with a 64-channel head coil. Functional and diffusion-weighted images are acquired using the Minnesota CMRR multiband accelerated pulse echo planar imaging (EPI) sequences [57]. For functional imaging, a T2*-weighted EPI sequence with repetition time (TR) of 800 ms, echo time (TE) of 37 ms, flip angle of 52°, 72 axial slices, 2-mm slice thickness, field of view (FoV) of 208 mm (whole-brain coverage was ensured by tilting the FoV to the individual anterior commissure – posterior commissure line) was used. Diffusion tensor imaging data are acquired using an EPI sequence with the following parameters: TR of 2100 ms, TE of 62 ms, flip angle of 72°, 92 slices, 1.50-mm isotropic resolution slice thickness, FoV of 210 mm, and 64 diffusion directions at *b* value of 1500 s/mm^2^. For anatomical images, a T1-weighted magnetization-prepared rapid gradient-echo sequence with the following parameters: TR of 2500 ms, TE of 2.22 ms, 208 axial slices, 0.8-mm slice thickness, FoV of 256 mm will be used. Furthermore, to assess the stroke area, a standard Siemens Prisma T2-weighted fluid-attenuated inversion recovery sequence with TR of 9000 ms, TE of 81 ms, 36 axial slices, 4-mm slice thickness, and FoV of 230 mm will be used.

### Working Memory Task

The working memory task will be a modified version of a well-known Sternberg task [58-62] with 4 task conditions. Given that our study population comprises older patients with stroke with PSD and without PSD, further modifications of a previously published Sternberg task [58,59,61,62] were necessary. Previous studies have reported difficulties regarding the feasibility of performing a working memory task after delirium [28,32], which we have taken into consideration. To enhance feasibility, the following modifications were implemented: [1] reduction of task difficulty by reducing the number of stimuli in the memory set from 5 to 4 items [2], shortening of the total task duration [3], adjustment of experimental conditions by including objects to enable the examination of the visual-spatial components of working memory.

**Figure 2.**
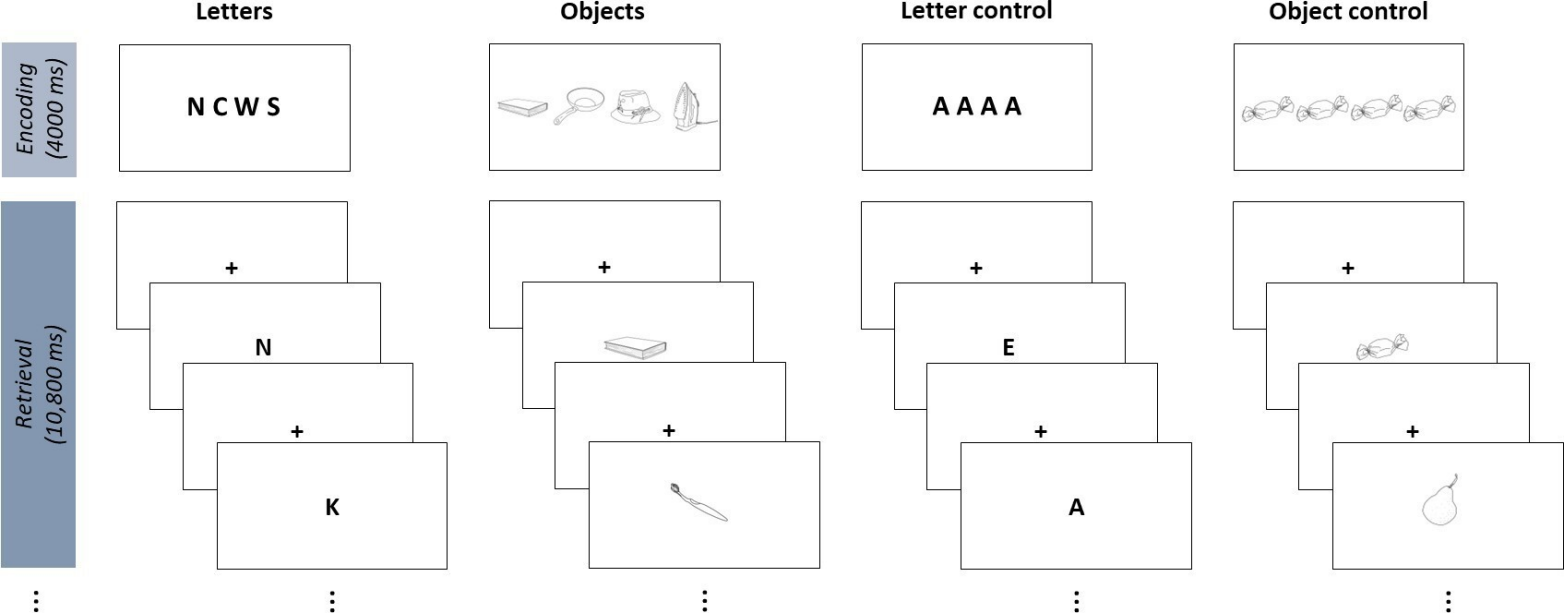
Modified version of the Sternberg task (working memory task) with 4 task conditions (letters, letter control, objects, and object control) each consisting of an encoding and a retrieval phase.

This modified version of the Sternberg task consists of 4 task conditions with different working memory categories, that is, verbal and visual items. In detail, 2 conditions comprise letters (letter condition [LE] and letter control condition [LC]) and 2 comprise objects (object condition and object control condition), as illustrated in Figure 2. The letters in the letter condition (LE) consist of consonants, and the letters in the control condition (LC) consist of vowels. In order to map the differences in letters (eg, FGMP, as in the letter conditions) onto the differences in objects, several semantic categories were selected for the object conditions, for example, pictures of daily life items, clothes items, kitchen tools, and electrical devices. All objects were derived from the bank of standardized stimuli line drawings [63,64]. To minimize condition-dependent effects such as differences in visual activations, eye movement, or spatial attention, all stimuli are presented on a white screen, at a fixed position and in black font or black line drawings (Figure 2). All conditions are presented 5 times in a pseudo-randomized and counterbalanced order. The experiment will be performed using the Presentation software (version 23.0; Neurobehavioral Systems, Inc) [65].

Each trial consists of an encoding phase and a retrieval phase. During the encoding phase, a set of 4 letters or objects (memory set) is presented and has to be memorized. After a variable interstimulus interval (1000‐1600 ms, randomly jittered in steps of 200 ms), 4 single-letter probes (2 targets, 2 nontargets) are presented for 1800 ms each in the retrieval phase (each followed by a fixation cross for 1200 ms), and the patient has to indicate after each single-letter probe whether the probe was part of the previously shown memory set or not. Patients have to press the left button on an MRI-compatible response pad to indicate that the single-letter probe was part of the memory set or press the right button if it was not. The total duration time of the working memory task is approximately 6.45 minutes. Figure 2 illustrates the conditions and trials of the working memory task.

### Statistical Analysis

#### Multicomponent Neuropsychological Assessment

In general, analysis will be conducted using SPSS Statistics (version 28.0.0.0; IBM Corp) [66] and MATLAB (R2024a; MathWorks Inc) [67].

In a first step, the analysis of the abovementioned tests, questionnaires, and clinical scores will be conducted according to the applicable manual. Raw scores will be transformed into norm-referenced *z* scores if standardizations are available.

In the second step, for the assessment of PSCI, a global and a domain-specific PSCI score will be defined in accordance with the guidelines of the International Society for Vascular Behavioral and Cognitive Disorders [68] and similarly to previous studies [39,69-72]. Further, the distinction between average cognitive functioning and mild, moderate, or severe cognitive impairment will be made according to the performance level on norm-referenced *z* scores, which is in line with the recommendations of multiple neuropsychological societies [73]. In detail, PSCI is a 5-level performance score. Performance level within 1 SD below or above the mean is defined as average cognitive functioning (PSCI=0). A range between 1 and 2 SDs below the mean (between third and 16th percentile) on at least 1 cognitive domain is defined as mild cognitive impairment (PSCI=1). A performance level 2 SDs below the mean (lower than the third percentile) in at least 2 domains is defined as moderate (PSCI=2), while a performance level 3 SDs below the mean (lower than the first percentile) in at least 2 cognitive domains or 2 SDs below the mean in most of the domains is defined as severe cognitive impairment (PSCI=3). Due to increased mortality in the PSD group, and to accommodate clinical realities as well as access feasibility of our study protocol, we use an intention-to-treat analysis for the primary end point. Therefore, we have added a level (PSCI=4), which reflects the death of a patient before the 3-month follow-up session. The following domains are defined: attention and information speed, executive functions, verbal memory, language, visuospatial construction, and orientation (Table 1). If a cognitive domain is represented by more than 1 test, the mean of the *z* scores will be used. We do not apply explicit weighting to individual test components when creating the composite scores for global and domain-specific poststroke cognitive function to ensure that each test within a domain contributes equally to the composite score, maintaining consistency with established neuropsychological standards. The primary endpoint will be a between-group comparison of the cognitive outcome, defined as global PSCI at a 3-month follow-up (S3). The groups will also be balanced for age and sex. Secondary outcome measures will be a between-group comparison of the domain-specific PSCI at a 3-month follow-up (S3).

Further, multivariate linear regression models will be used to analyze multi-item prognostic or predictive tools for cognitive outcome (eg, global PSCI) at the follow-up measurement (S3) by multimodal parameters assessed during the acute phase poststroke (S1), such as NIHSS, acute treatment, and working memory performance during fMRI or fMRI connectivity measures. As some tests will be digitalized using a tablet and a digitizer pencil, new parameters representing search behavior, for example, time on surface and time in air [42,74], will be recorded and might help to differentiate between PSD and non-PSD groups or identify patients with stroke with a high delirium risk.

Missing data will be handled in the following manner. For missing data in a 2-test subdomain, we plan to use the *z* score of the other test within the same subdomain. For missing data in a single test subdomain, we plan to use a multiple imputation approach or maximum likelihood estimation.

#### Neuroimaging Data

The preprocessing of the structural and functional data will be performed using standard routines of the Statistical Parametric Mapping software (SPM12) [75] within MATLAB [67]. In the first step, lesions are delineated on T2-weighted (fluid-attenuated inversion recovery) or T1*-weighted (T1-weighted magnetization-prepared rapid gradient-echo sequence) images. In the second step, the raw data will be preprocessed including the following steps: realignment, slice timing, coregistration, normalization, and spatial smoothing. This step includes the lesion correction during the normalization process to ensure accurate spatial alignment across subjects, using standard delineation and lesion correction methods [76-78]. In the third step, first-level analysis will be conducted using a general linear model including the following 8 task regressors: 4 regressors for each condition (ie, LE, LC, object condition, and object control condition) in the encoding phase and 4 regressors for each condition (ie, LE, LC, object condition, and object control condition) in the retrieval phase. These regressors will be modeled using a delta (stick) function for the encoding phase (to best account for phasic changes in the neural response to the target set) and a boxcar function for the retrieval phase. Besides brain activation, we also plan to analyze functional connectivity within the frontal-parietal networks or ascending reticular activating system. For the diffusion tensor imaging data, fractional anisotropy, axial or radial diffusivity, mean diffusivity, and so forth will be used to analyze differences in structural connectivity.

For the retrieval phase of the working memory task, the mean reaction time for target and nontarget probes will be calculated for all the 4 task conditions. Accuracy will be calculated for each condition as a percentage of correctly identified probes. Presentation (version 23.0; Neurobehavioral Systems, Inc) [65] will be used for calculating these performance parameters. Further analysis steps will be conducted using SPSS Statistics (version 28.0.0.0; IBM Corp) [66] and MATLAB (R2024a) [67], for example, to compare the performance parameters of the task conditions between the PSD and non-PSD group, for example, multifactor ANOVA.

## Results

This study was funded in December 2023. The recruitment of patients was initiated in June 2024 and will continue until September 2025. As of May 2025, 15 patients with stroke have been included. Data acquisition will continue until December 2025. Data analysis will commence at the beginning of 2026, and study findings are expected to be published in summer 2026.

## Discussion

Here, we present the protocol of a longitudinal study (RECOVER) designed to investigate both short-term and long-term effects of PSD on cognitive impairment and recovery in patients with stroke. PSCI is assessed using a multimodal approach that includes a multicomponent digital neuropsychological assessment and task-based fMRI at 3 time points: during the acute (prior PSD development) phase, the postacute phase, and at the 3-month follow-up.

Prior behavioral studies showed significantly poorer cognitive outcome (eg, in global cognition or memory decline) in both patients with stroke with PSD and without PSD compared to healthy controls [1,24,33]. Thus, a clear distinction between “stroke-related” and “PSD-related” cognitive impairments has not been established so far.

Brain neuroimaging studies using structural MRI reported dysconnectivity within the cortical-subcortical systems, for example, reticulo-thalamo-cortical network [1,13,79] in patients with delirium. fMRI studies using resting state have further revealed disrupted connectivity in dorsal-prefrontal and subcortical networks in patients with delirium (compared to healthy controls). These alterations may be linked to core delirium symptoms, such as disorientation, altered arousal, attention deficits, or disturbances in the sleep-wake cycle [1,13,16,28,30,80,81].

Memory is a fundamental aspect of human cognition and a frequently affected domain in patients with stroke with PSD and without PSD [22]. However, memory impairments may not be detectable during resting-state fMRI [31]. Unfortunately, task-based fMRI studies that allow for the direct assessment of memory functions are currently very limited. To date, only 1 fMRI study has investigated this challenging area, reporting no significant association between delirium and brain region activity [32]. However, this study raises methodological concerns due to the absence of a control group and the use of an infeasible working memory task. Further research is therefore needed, alongside the development of an adequate and reliable assessment of working memory during fMRI.

Finally, there remains a notable gap in the literature regarding appropriate methods for studying PSD and related cognitive outcomes. This gap has been addressed by this longitudinal study protocol, which includes 3 measurement points and 2 groups of patients with stroke, PSD, and non-PSD (control), to assess PSCI. We followed a multicomponent assessment strategy for the neuropsychological assessment and introduced a suitable digital tablet app tailored for both patients with stroke with PSD and without PSD. This partly tablet−based neuropsychological assessment enables the generation of additional neuropsychological parameters, such as time in air or time on surface [42,74], which may help to detect patients with PSD at an early stage.

In addition, we present an extended (ie, verbal and visual items) and modified version of a well-known working memory task (Sternberg task) [58-61] to examine patients with stroke with PSD and without PSD. This study design enables the direct assessment of memory functions at both the behavior level via neuropsychological testing and advanced parameters derived from digitalized tests, and at the brain level via task-based fMRI, where patients actively perform the working memory task during scanning. Therefore, we can map the neural correlates of working memory functions and conduct brain-behavior correlation analyses. Additionally, this comprehensive longitudinal design, involving 2 patient groups (PSD and non-PSD), may allow us to differentiate between stroke-related and PSD-related cognitive impairments. Moreover, we aim to contribute preliminary insights toward developing protective and prognostic tools that support the early detection and treatment of PSD, ultimately helping to improve long-term cognitive recovery in patients with stroke.

The findings and experience gained from this proof-of-concept protocol will serve as early, preliminary investigation and provide essential groundwork for the design and implementation of future multicenter studies with a larger sample size. This protocol represents a first step toward expanding previous knowledge and improving the understanding of the contribution of PSD to long-term cognitive impairment after stroke.

These early findings from the proof-of-concept study hold promising clinical implications for the diagnosis and treatment of PSD, with further research required to validate these results on a larger scale. A more profound understanding of the cognitive dysfunctions associated with PSD could significantly influence clinical decision-making and rehabilitation strategies. Recognizing PSD as a distinct clinical condition characterized by specific patterns of cognitive impairment could help clinicians differentiate it from typical poststroke cognitive dysfunction. Specifically, the early identification of PSD-related cognitive deficits may enable the timely initiation of targeted, high-intensity cognitive rehabilitation, including domain-specific interventions such as those focused on attention, memory, and executive function. This approach has the potential to guide the development of individualized care plans tailored to patients’ needs, prevent long-term cognitive decline, and promote optimal cognitive recovery.

However, this study protocol has several limitations. First, our study population consisted of patients with stroke with mild-to-moderate symptoms (NIHSS ≤15) and excluded patients with stroke with more severe stroke symptoms, particularly those with a GCS score ≤8, as well as patients with aphasia, which limits the generalizability of our findings to the broader population with stroke. However, patients with global aphasia are often unable to comprehend test instructions or follow simple requests (eg, “close your eyes”), making the assessment of cognitive functions, such as memory functions, extremely challenging, time-consuming, and often frustrating for the patient. Patients with a GCS score ≤ 8 have typically significantly reduced levels of consciousness, making a reliable cognitive assessment not possible. Second, the sample size is 40. Although this proof-of-concept study is underpowered, it is essential for gathering preliminary data and assessing feasibility. Insights gained will provide critical groundwork for designing larger, adequately powered studies in the future. Another important limitation of our study protocol is the variable timing of assessments, especially for S2, which is scheduled within a broad window of 5-14 days post stroke. These flexible time windows were intentionally selected to accommodate clinical realities and to minimize dropout rates and missing data. Regarding S2, this variability is driven by clinical realities, including patient transfers on short notice, fluctuating medical conditions, limited MRI availability, and the unpredictable course of delirium recovery. Unfortunately, the MRI assessment cannot take place during the acute phase of a delirium but only after partial recovery from delirium, a common problem in behavioral and neuroimaging studies on delirium or PSD. While this flexible scheduling approach enhances the feasibility of conducting comprehensive neuropsychological and imaging assessments in a population with acute stroke, it introduces potential confounding effects related to differences in the timing of measurements across patients. To mitigate this issue, we will incorporate the number of days post stroke as a covariate in our statistical analyses. Future large-scale studies should consider using more standardized normalization strategies to handle timing variability at S2, such as time normalization or statistical correction for nonlinear recovery effects in mixed effects models.

Another concern in our multimodal longitudinal study is the increased risk of participant dropout compared to cross-sectional studies. This risk is particularly pronounced among patients with PSD, given the association between PSD, lower quality of life, and higher mortality rates, which may lead to a higher than anticipated dropout rate. To address this, we have implemented several mitigation strategies. First, as described above, we have established predefined time windows to accommodate clinical realities within this vulnerable study population while prioritizing patients’ well-being. Assessments are adjusted within predefined time windows based on the patient’s condition, balancing protocol adherence with clinical care and comfort. Second, both neuropsychological and MRI assessments include opportunities for breaks or pauses to manage patient fatigue or distress. Third, when the patient’s condition or transfer prevents in-hospital assessment, we offer follow-up visits at patients’ homes or rehabilitation centers. Collectively, these measures enhance feasibility and data completeness while ensuring patient safety and comfort.

This study protocol involves important ethical considerations due to the vulnerability of patients with acute stroke, particularly those experiencing PSD. Ensuring ethical responsibility and patient safety is of the utmost importance. In an effort to minimize the burden on participants, both neuropsychological testing and MRI sessions are kept as brief as possible and can be paused or rescheduled based on the patient’s condition. Testing is immediately halted if any signs of distress or fatigue arise. Throughout the entirety of the procedures, the presence and attentiveness of clinical staff are maintained. The inclusion of cognitively vulnerable patients is ethically justified, as this group is often underrepresented in research. However, their participation is carefully balanced with stringent safeguards to prevent any undue burden on their health or recovery. The presented protocol is critical to provide a comprehensive framework for assessing cognitive outcomes tailored to the challenges of evaluating older patients with stroke with and without PSD.

While this was beyond the scope of this proof-of-concept study, future studies should be initiated as multicenter trials to enhance the generalizability and robustness of the findings. Increasing the sample size will be essential to achieve adequate group sizes and improve statistical power. Additionally, balancing groups according to key variables, such as lesion location or stroke severity, would strengthen the validity of comparisons.

Another important aspect is the consideration of the amount of cognitive rehabilitation on PSCI. Unfortunately, this was beyond the scope of this study, and this protocol includes only documentation of whether rehabilitation was received and its duration. A detailed evaluation of the effect of cognitive rehabilitation impact on long-term outcomes should be addressed in future studies. Another consideration would be extended follow-ups, for example, after 6 and 12 months, to better capture cognitive trajectories.

This protocol represents a proof-of-concept study designed to systematically and comprehensively examine cognitive functions in critically ill patients with acute stroke with and without delirium. It combines comprehensive neuropsychological testing with a multimodal MRI protocol, including digitalized assessments and task-based functional MRI. By addressing a significant clinical gap and using innovative methodologies, this study lays essential groundwork toward larger, adequately powered studies.

## Abbreviations

EPI: echo planar imaging sequence
fMRI: functional magnetic resonance imaging
FoV: field of view
ICDSC: Intensive Care Delirium Screening Checklist
LC: letter control condition
LE: letter condition
MRI: magnetic resonance imaging
NIHSS: National Institutes of Health Stroke Scale
PSCI: poststroke cognitive impairment
PSD: poststroke delirium
RECOVER: Cognitive Recovery After Poststroke Delirium
TE: echo time
TR: repetition time
TuCAN: Tübingen Cognitive Assessment for Neuropsychiatric Disorders
